# Biological impacts of rising temperatures on maternal, fetal and newborn health: protocol for a cohort study (BIRTH-Cohort)

**DOI:** 10.1136/bmjopen-2025-107773

**Published:** 2026-03-18

**Authors:** Jai K Das, Asma Abdul Malik, Aris T Papageorghiou, Suneel Piryani, Mahnaz Ambareen, Nadeem Zuberi, Zaheena Islam, Nuruddin Mohammed, Narjis Fatima Hussain, Zainab Samad, Farhala M Baloch, Saqib Qazi, Arjumand Rizvi, Imran Ahmed Chauhadry, Junaid Iqbal, Shazia Samad Mohsin, Fatima Ali, Lumaan Sheikh, Kehkashan Begum, Rabia Zuberi, Simon Cousens, Stephen Kennedy, Zulfiqar Ahmed Bhutta

**Affiliations:** 1 Institute for Global Health and Development, The Aga Khan University, Karachi, Sindh, Pakistan; 2 Department of Paediatrics and Child Health, The Aga Khan University, Karachi, Sindh, Pakistan; 3 Nuffield Department of Women’s & Reproductive Health, University of Oxford, Oxford, Oxfordshire, UK; 4 Oxford Maternal and Perinatal Health Institute, Green Templeton College, University of Oxford, Oxford, Oxfordshire, UK; 5 Department of Obstetrics and Gynaecology, The Aga Khan University Hospital, Karachi, Sindh, Pakistan; 6 Centre for Global Child Health, The Hospital for Sick Children, Toronto, Ontario, Canada; 7 Department of Medicine, The Aga Khan University Hospital, Karachi, Sindh, Pakistan; 8 Section of Pediatric Surgery, Department of Surgery, The Aga Khan University Hospital, Karachi, Sindh, Pakistan; 9 Centre of Excellence in Women and Child Heath, The Aga Khan University, Karachi, Pakistan; 10 Nutrition Research Laboratory, The Aga Khan University, Karachi, Sindh, Pakistan; 11 Division of Cardiothoracic Sciences, Sindh Institute of Urology and Transplantation, Karachi, Sindh, Pakistan; 12 Infectious and Tropical Diseases, London School of Hygiene and Tropical Medicine, London, UK

**Keywords:** Pregnancy, Climate Change, Reproductive medicine, Observational Study, PUBLIC HEALTH

## Abstract

**ABSTRACT:**

**Introduction:**

Climate change has led to extreme heat events, disproportionately affecting vulnerable populations. Heat stress during pregnancy is linked to adverse health outcomes, yet the biological mechanisms remain poorly understood. This research study aims to investigate the effect of environmental heat on maternal, fetal and infant health and examine the biological pathways linking heat stress to adverse pregnancy outcomes.

**Methods and analysis:**

This prospective cohort study will recruit 6000 pregnant women from three districts in Sindh, Pakistan. Eligible participants ≥18 years old, will have a minimum of five scheduled visits from <14 weeks’ gestation and will be followed up to 12 months postpartum. Primary outcomes include low birth weight and small vulnerable newborns (SVN); secondary outcomes include preterm birth, small for gestational age (SGA), miscarriage, stillbirth, and composite maternal and neonatal morbidity and mortality. Fetal ultrasound scans with Doppler assessments will be performed at each visit to measure fetal growth, uteroplacental and fetoplacental circulation. Each woman’s heat exposure will be measured using wearable sensors and heat strain biomarkers. In a subset of 1000 women, maternal heart rate, skin temperature sleep patterns and physical activity will be monitored throughout pregnancy using wearable devices. Time-varying, distributed lag and non-linear models will examine associations between heat stress indices and pregnancy outcomes.

**Ethics and dissemination:**

The study has received ethical approval from the Aga Khan University (AKU) (Ref: 26249) and the Pakistan National Bioethics Committee (Ref: 1065/23/1736). Written informed consent will be obtained from all participants before enrolment. Referral pathways to healthcare facilities will be established to ensure timely management of pregnancy complications. Findings will be disseminated through peer-reviewed publications, scientific conferences, and engagement with policymakers and public health stakeholders to inform climate-resilient maternal health strategies. Results will also be shared with participants and communities through meetings and informal sessions to raise awareness and support evidence-based heat adaptation.

**Trial registration number:**

NCT01234567.

**STRENGTHS AND LIMITATIONS OF THIS STUDY**

- First large-scale prospective cohort in Pakistan to investigate the association between heat stress and pregnancy and birth outcomes.
- Use of personal wearable devices will allow to measure individual exposures and physiological profiling.
- Serial fetal biometry and Doppler assessments will allow antenatal classification of fetal growth restriction (FGR) phenotypes, rather than relying solely on birth weight as a proxy for impaired fetal growth.
- Collection of cord blood, placental tissue, and archived stool, nasopharyngeal, and serum samples will allow transcriptomic and epigenetic analyses in the future.
- We anticipate challenges in compliance with wearable device use due to sociocultural acceptability and device discomfort during hot weather.

## Introduction

### Epidemiology and burden

 Anthropogenic climate change has led to rising global temperatures and an increase in the frequency, duration and intensity of extreme heat events.^1 2^ Women and children, especially in low-resource settings, are disproportionately affected due to limited adaptive capacity and socioeconomic challenges.^3^,^6^ South Asia is particularly vulnerable to extreme heat due to socioeconomic factors, such as poverty, illiteracy, high population density, unfavourable infrastructure and limited adaptive capacity.^7^,^10^ Pakistan faces severe heat stress, with high-intensity heatwaves lasting up to 41 days annually and recorded temperatures exceeding 53.7°C. Heat extremes worsen water scarcity, food insecurity and climate-driven migration, especially in rural Sindh and Balochistan.^11^

Pregnancy is a critical period marked by extensive metabolic and physiological changes in the mother and developing fetus.^12^ Exposure to environmental stressors, such as heat stress, can disrupt tightly regulated biological processes, leading to maternal and fetal physiological strain.^13^ This disruption may result in immediate adverse effects and long-term health consequences for both mothers and their children.^14^,^18^ Epidemiological studies link heat stress to adverse pregnancy outcomes, including pre-eclampsia, gestational diabetes, hypertensive disorders and cardiovascular complications.^519^,^21^ It is also associated with adverse birth outcomes, such as preterm, low birth weight (LBW), and small-for-gestational-age (SGA) births, and stillbirth.^22^,^24^ A recent systematic review of 198 studies across 66 countries from diverse settings found that a 1°C rise in ambient temperature increased the odds of preterm birth by 1.04 (95% CI 1.03 to 1.06), and by 1.26 (95% CI 1.08 to 1.47) during heatwaves. It reported a 1.13-fold (95% CI 0.95 to 1.34) increase in stillbirth, and the odds of any obstetric complication increased by 1.25 (95% CI 1.09 to 1.42) during heatwaves.^25^ The evidence further suggests that these associations are largest among women in the lowest socioeconomic and most vulnerable groups.^26^

### Proposed pathophysiological mechanisms

Despite mounting epidemiological data, empirical evidence on the underlying pathophysiological mechanisms and the causal role of heat exposure remains poorly understood, with most evidence originating mainly from high-income countries.^27^ While the precise mechanisms remain unclear, several potential pathways have been proposed. Pregnant women typically maintain adequate thermoregulatory capacity; however, extreme heat exposure can overwhelm these mechanisms, leading to heat strain—the physiological response to heat stress.^21^ While the precise mechanisms remain unclear, several potential pathways have been proposed.

A maternal core temperature rise of 1.5–2°C above baseline (~39°C) is teratogenic, particularly in the first trimester, and is associated with neural tube defects and orofacial anomalies.^28^,^30^ Animal studies suggest that heat stress may promote oxytocin secretion and prostaglandin F2-alpha (F2α) release during the third trimester and increase the risk of preterm birth.^31 32^ Heat stress is also linked to oxidative stress, elevated cortisol, cytokines and maternal heat shock proteins, such as heat-shock protein-70 (HSP-70), which is associated with fetal growth restriction (FGR), pre-eclampsia and preterm birth.^33^ It may impair placental vascular development, contributing to placental insufficiency and FGR, and may be an indicator of maternal cardiovascular risk.^34^ Evidence from non-pregnant adults shows that heat strain activates coagulation pathways,^35^ raising concerns of thromboembolic risk during pregnancy.^36^

**Figure 1:**
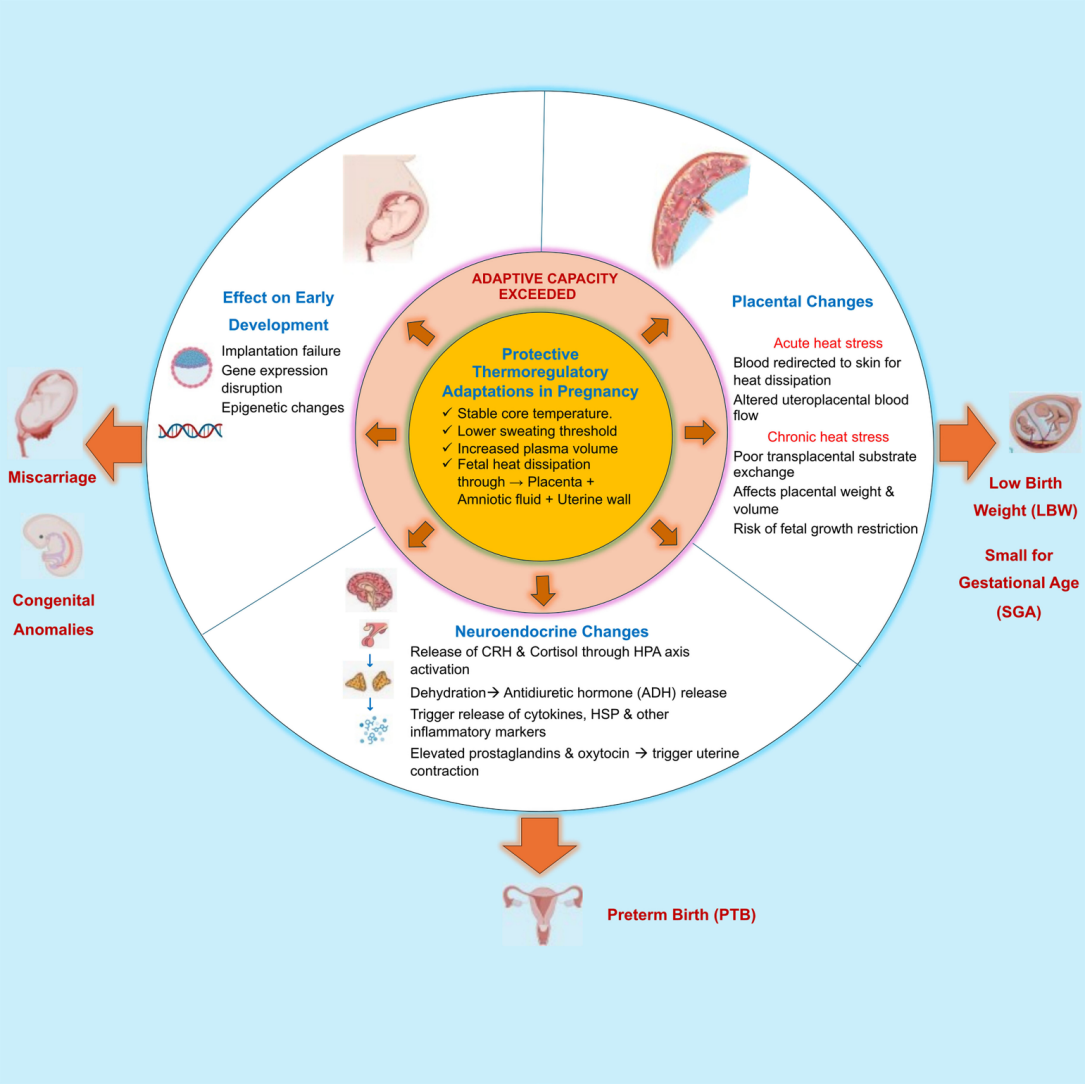
Conceptual framework of the impact of heat stress on maternal, fetal and birth outcomes. HSP, Heat-shock protein; CRH, Corticotropin-releasing hormone; HPA, Hypothalamic-Pituitary-Adrenal axis.

Figure 1 presents a conceptual framework that illustrates how exceeding adaptive capacity to heat stress can trigger multiple biological pathways, leading to adverse maternal and birth outcomes. These include: (1) early pregnancy disruptions, such as implantation failure, gene expression changes and miscarriage; (2) placental insufficiency due to altered blood flow and reduced transplacental exchange from acute and chronic heat exposure, contributing to FGR; and (3) neuroendocrine activation, including increased cortisol, antidiuretic hormone, inflammatory mediators and prostaglandins that may trigger uterine contractions.

During pregnancy, plasma volume and cardiac output increase by 50% to support placental perfusion,^37 38^ while extreme heat exposure may redirect blood flow to the skin, reducing fetal oxygen and nutrient supply. However, animal studies suggest increased uterine blood flow during exogenous heat stress, and evidence in humans remains limited and inconsistent. Dehydration, a common consequence of heat exposure, may exacerbate these risks by reducing uterine blood flow and stimulating prostaglandin release, thereby increasing the likelihood of preterm birth.^39^

Most evidence on the effects of extreme heat originates from the Global North,^2^ with limited data from low-income and middle-income countries (LMICs), where preterm birth and stillbirth rates are among the highest globally. In rural areas of Pakistan, rising temperatures are compounded by extreme poverty, food insecurity, subsistence agriculture and outdoor labour.^40^ Pregnant women in these settings are particularly at risk as they often continue to engage in physically demanding work throughout pregnancy, compounding their exposure to heat stress.^41^ This research study aims to address critical knowledge gaps by investigating the impacts of extreme heat on maternal, fetal and infant health and explore the biological pathways and mechanisms underlying these effects across different stages of pregnancy.

## Methods and analysis

### Study objectives

The primary objectives of the study are:

- To assess the acute and sustained effects of environmental heat exposure on pregnancy and birth outcomes in rural and peri-urban areas of Pakistan.
- To assess the bio-physiological pathways through which heat stress affects maternal, fetal and infant health across different gestational ages in rural and peri-urban areas of Pakistan.

The secondary objectives are:

- To evaluate how gestational age, sociodemographic, maternal and nutritional factors modify the association between heat exposure and adverse pregnancy outcomes in rural and peri-urban areas of Pakistan.

### Study design and settings

This prospective cohort study will enrol women who are less than 14 weeks pregnant and monitor them until delivery. Each mother-infant pair will then be followed monthly for 12 months after delivery. The study will be conducted in the three rural/peri-urban districts of the province of Sindh in Pakistan: Matiari, Tando Muhammad Khan (TMK) and Tharparkar. Sindh is the southeastern region of the country and is Pakistan’s second most populous province. The selected districts are predominantly rural, interspersed with peri-urban areas. The districts of Matiari and TMK are located in central Sindh and experience a subtropical climate characterised by extremely hot summers with temperatures often exceeding 40°C. Mithi, the administrative capital of Tharparkar district, has an arid climate marked by prolonged periods of intense heat. The temperature during the peak summer months frequently exceeds 45°C, particularly in June, the hottest month of the year. The winter season in these districts lasts approximately 3–4 months, with January being the coldest month, and average temperatures ranging from 12°C to 24°C.^42^,^44^

Underprivileged populations in these districts face significant challenges, including poverty, lack of education, food insecurity and limited access to healthcare.^45 46^ Women in rural Sindh constitute over 65% of the agricultural and livestock management workforce. They engage in intensive manual labour such as crop production, sowing, weeding, harvesting, post-harvest processing, and caring for livestock and poultry. In addition to these outdoor activities, many women spend long hours indoors cooking over open stoves and walking long distances to fetch water and firewood. Most villages lack basic infrastructure, such as a reliable electricity supply, which limits access to cooling appliances and increases residents’ vulnerability to extreme temperatures.^45 46^ Many women work up to 15 hours a day including during pregnancy, increasing their susceptibility to heat stress.^1 47 48^

### Study population and eligibility criteria

The study will enrol pregnant women in their first trimester residing in peri-urban and rural areas of the three districts.

#### Inclusion criteria

- Live pregnancy.
- Gestational age less than or equal to 13 weeks and 6 days at enrolment.
- At least 18 years of age.
- Permanent resident of the study district with plans for delivery within the district.
- Willing to visit the study research centre for assessments throughout pregnancy and able to comply with all study requirements.

#### Exclusion criteria

- Plan to relocate outside the district for more than 3 months during the study period or for delivery.

### Study outcomes

The primary and secondary outcomes will be assessed across the entire cohort of pregnant women, while intermediate outcomes will be assessed in a subset of women through in-depth data collection.

#### Primary outcomes

The primary outcomes of the study are:

1. LBW: birth weight <2500 grams (5.5 pounds).
2. Small vulnerable newborns (SVN): all live newborns who are either preterm, SGA or LBW.^49^

#### Secondary outcomes

The secondary outcomes of the study are:

1. Preterm birth: live birth <37 weeks’ gestation. It will be further categorised by gestational subgroups: extreme preterm (<28 weeks), very preterm (28–31 weeks), moderate preterm (32–33 weeks) and late preterm (34–36 weeks).
2. SGA: neonates born with birth weight <10th centile for gestational age and sex based on the International Fetal and Newborn Growth Consortium for the 21st Century (INTERGROWTH-21st) standards.^50^ We define term-SGA as a baby born SGA at ≥37 weeks of gestation, and preterm-SGA as infants born SGA at <37 weeks of gestation.^51^
3. Miscarriage: fetal loss before 22 weeks’ gestation.
4. Stillbirth: baby born with no signs of life at or after 22 weeks’ gestation (a 28-week cut-off will also be used for international comparison).^52^
5. Gestational hypertension: blood pressure >140/90 mm Hg, first measured after 20 weeks’ gestation in a previously normotensive woman. Pre-eclampsia will be defined as gestational hypertension accompanied by proteinuria, indicated by a urine dipstick protein reading of >2+.^53^
6. Gestational diabetes mellitus (GDM): ^54^ Pregnant women screened at 28 weeks with a 50 g Glucose Challenge Test (GCT) with a plasma glucose level of ≥130 mg/dL one hour after the GCT will undergo a 75 g Oral Glucose Tolerance Test (OGTT) the next day. Since the GCT does not require fasting, it serves as the initial screening step for logistical convenience. GDM will be diagnosed based on the IADPSG/WHO criteria, requiring any of the following plasma glucose thresholds during the OGTT: fasting ≥92 mg/dL, 1-hour ≥180 mg/dL, or 2-hour ≥153 mg/dL.
7. Composite and individual maternal morbidity and mortality (CMMM) outcomes defined as the presence of any of the following: postpartum haemorrhage (PPH), chorioamnionitis, endometritis, transfusion of ≥2 units of blood during pregnancy or post-partum (within 6 weeks after birth), intensive care unit (ICU) admission, venous thromboembolism, uterine rupture, hysterectomy, cystotomy, ureteral or bowel injury, or maternal death.^55^
8. Composite and individual adverse pregnancy outcomes for the newborn,^56^ including any of the following,
  - FGR: we will classify FGR using the criteria set out in the international consensus definition.^57^ Early FGR (<32 weeks) will be defined using three parameters: abdominal circumference (AC) <3rd centile, estimated fetal weight (EFW) <3rd centile, and absent end-diastolic flow in the umbilical artery (UA), and two parameters: AC or EFW <3rd centile for late FGR (>32 weeks).
  - Pre-eclampsia with severe features: defined as repeated episodes of severe hypertension despite maintenance treatment with at least two (instead of three as per the International Society for the Study of Hypertension in Pregnancy (ISSHP) definition) antihypertensive agents, plus pre-eclampsia arising before 34 weeks’ gestation according to amended ISSHP criteria.
  - Neonatal death: defined as death <28 days after birth.
  - Clinical diagnosis of placental abruption.
  - Severe placental lesions: defined as high-grade or severe maternal vascular malperfusion; high-grade/severe fetal vascular malperfusion.
9. Maternal gestational weight gain (GWG): assessed according to the Institute of Medicine (IOM) guidelines^58^ using first-trimester body mass index (BMI) as a baseline to classify GWG as inadequate, adequate or excessive.
10. Any congenital fetal anomalies.
11. Infant and Young Child Feeding (IYCF): assessed post-delivery using the 2021 WHO/UNICEF IYCF indicators through 24-hour dietary recalls with the mothers.^59^
12. Length-for-age Z score (LAZ) and weight-for-age Z score (WAZ): measured post-delivery to assess monthly infant growth, following the INTERGROWTH-21st standards.^50^

#### Intermediate outcomes

In addition, the study will assess intermediate outcomes for a subset of 1000 women through continuous monitoring of detailed physiological parameters throughout pregnancy, including:

- Fetal echocardiogram (ECHO) parameters.
- Skin temperature using Thermochron i-Buttons.
- Heart rate, daily activity levels and sleep patterns measured using the Fitbit smartwatch.

### Sample size

To detect a 15% increase in the risk of LBW among pregnant women exposed to heat stress compared with an unexposed cohort (1:2 ratio), assuming 80% power, a 5% significance level, and a 20% attrition rate, 6000 participants will be required. This calculation is based on a baseline LBW prevalence of 26.2% reported in the 2018 National Nutrition Survey for the rural population. The sample size for SVN under the same assumptions is 2106 pregnant women based on a prevalence of 46%.^60^

For the detailed substudy, sample size calculations were based on various key physiological parameters assessed in controlled settings, including changes in skin temperature, maternal heart rate, blood pressure, cardiac output and fetal heart rate. To detect a 10% change in these parameters, the maximum required sample size is 200 participants, assuming 90% power, a 5% significance level and a 20% attrition rate. However, due to the limited scope of previous studies conducted under controlled conditions and the anticipated higher attrition rate, we have chosen a substudy sample size of 1000 women.

### Recruitment and training of the study staff

We will recruit experienced local staff, including doctors, sonologists, nurses/midwives, and data and supervisory personnel. Study doctors will be qualified physicians with at least 2 years’ experience in obstetric ultrasound. They will receive additional training in ultrasound and fetal echocardiography through a 3-month programme at the Aga Khan University (AKU), along with completion of the International Society of Ultrasound in Obstetrics and Gynecology (ISUOG) ultrasound course.^61^ At each site, three teams (two data collectors and one supervisor each) will be hired. Central training will cover study protocols, detailed review of data tools and standardised anthropometry according to the Food and Nutrition Technical Assistance III Project (FANTA III) guidelines.^62^ Staff members will also receive training on the use of devices and data retrieval.

### Participant enrolment and data collection procedures

#### Identification and recruitment of the participants

Pregnant women will be identified through community outreach activities by study field staff in collaboration with lady health workers (LHWs). Teams will conduct pregnancy screening and participant tracking to enhance early recruitment while fostering community engagement and initiating discussions about the study before enrolment. Field staff will identify potential pregnancies, focusing on women who have recently married, had a gap of 1–2 years since their last pregnancy, or have expressed intentions to conceive. Field staff will use pregnancy test kits to facilitate early detection of pregnancies. Study doctors will perform ultrasound at the research centre to confirm pregnancy and determine gestational age for eligibility assessment. On obtaining written informed consent, a recruitment form will be completed. Study research centres will serve as focal points for participants’ antenatal assessments, ultrasound scans and biological sample collection. Free transportation to site offices will be provided. Deliveries will occur at participants’ chosen health facilities, as research centres will not offer delivery services.

#### Patient and public involvement

Participants will not be involved in the design, conduct, outcome selection or recruitment for this study. No patient or public involvement is planned during the implementation phase.

#### Data collection procedures

At enrolment (≤14 weeks of gestation), doctors at the research centre will confirm gestational age by ultrasound measurement of fetal crown rump length, with an accuracy of ±7 days following INTERGROWTH-21st standards^63^ and then conduct serial ultrasound and Doppler examinations, as well as fetal echocardiograms at designated time points during pregnancy. The data collection team will collect baseline information on demographics, socioeconomic status, work details, physical activity and household structure. Study doctors will collect data on past medical and obstetric history, current pregnancy status and antenatal care uptake, symptoms related to pregnancy complications (eg, bleeding, hypertension, fever), use of medications and supplements, health-seeking behaviours and sources of care. Anthropometric measurements, food insecurity^64^ and 24-hour dietary recalls will also be documented. Participants will be assessed at three additional time points until delivery: 18–22 weeks, 28–32 weeks and 36–37 weeks’ gestation.

#### Exposure ascertainment

The ambient temperature for each participant will be measured using wearable temperature and humidity loggers (Temp U) throughout the study period. The cohort subset will additionally be provided with a Fitbit smartwatch to monitor respiratory rate, heart rate, body temperature, sleep patterns and physical activity., Thermochron i-Button will be worn and placed in an abdominal belt to record skin temperatures continuously, with 0.1°C accuracy after calibration. This raw data will be used to calculate the heat stress indices.

#### Outcome ascertainment

Pregnancy-related outcomes will be ascertained and confirmed by study doctors through clinical examination at follow-up visits and laboratory confirmation. Labour details and outcomes related to CMMM will be obtained from medical record reviews. Birth weight, length and head circumference (HC) will be measured within 48 hours of delivery by trained data collectors (midwives) using standardised anthropometric tools and INTERGROWTH-21st standards. Study doctors and data collectors will undergo structured training sessions on standardised clinical assessment, data recording and anthropometric measurements.

#### Blood collection

At enrolment, a complete blood count (CBC) will be conducted for all participants (n=6000) to assess their baseline haematological status. Spot haemoglobin levels and urinalysis will be performed at all scheduled visits to monitor anemia, hydration status and screen for potential complications. In the subcohort (n=1000), additional serum biomarkers, including interleukin-6 (IL-6), soluble fms-like tyrosine kinase-1 to placental growth factor ratio (sFlt-1/PlGF), tumour necrosis factor-alpha (TNF-α) and HSP-70, will be measured at 28–32 weeks’ follow-up. Serum samples will be transferred into pre-labelled cryotubes and sealed in zip-lock bags. The samples will be temporarily stored in the field laboratory’s refrigerator and transported in temperature-controlled Coleman boxes at 2–8°C, equipped with digital temperature loggers for continuous monitoring. A summary of the planned biomarker assessments and their respective time points is provided in table 1.

**Table 1:** Imaging and biochemical assessments at different time points through pregnancy

Investigations		Enrollment before 14 weeks	18–22 weeks	28–32 weeks	36–37 weeks	Within 48 hours of birth
Viability scan and uterine artery Doppler	Imaging	✓				
Anomaly scan and uterine artery Doppler			✓			
Growth scan and multi-vessel Doppler of uterine & umbilical artery, middle cerebral artery				✓	✓	
Fetal Echocardiogram (ECHO)				✓		
Complete blood count (CBC)	Blood tests	✓				
Spot haemoglobin		✓	✓	✓	✓	
Fasting blood sugar (FBS)		✓		✓		
Interleukin-6				✓		
HSP-70				✓		
sFLT-1/PlGF				✓		
TNF-α				✓		
Urinalysis	Others	✓	✓	✓	✓	
Faecal sample & nasopharyngeal swab archiving for the subcohort		✓	✓	✓		
Placental and cord blood samples for the subcohort						✓

HSP-70, heat-shock protein-70; sFlt-1/ PlGF, soluble fms-like tyrosine kinase-1 to placental growth factor ratio; TNF-α, tumour necrosis factor-alpha.

#### Laboratory assessments

Spot haemoglobin levels will be measured with the HemoCue 301 colourimetry system (HemoCue AB, Ängelholm, Sweden). TNF-α and IL-6 will be analysed using the chemiluminescence immunoassay (CLIA) technique on the Snibe Maglumi X8 analyser. HSP-70 will be measured using ELISA microplate assays on the BioTek Epoch 2 Microplate Spectrophotometer. Urine analysis will assess kidney function and hydration status by measuring osmolality using freezing point depression and specific gravity via dipstick tests.

#### Ultrasound and Doppler imaging

**Figure 2:**
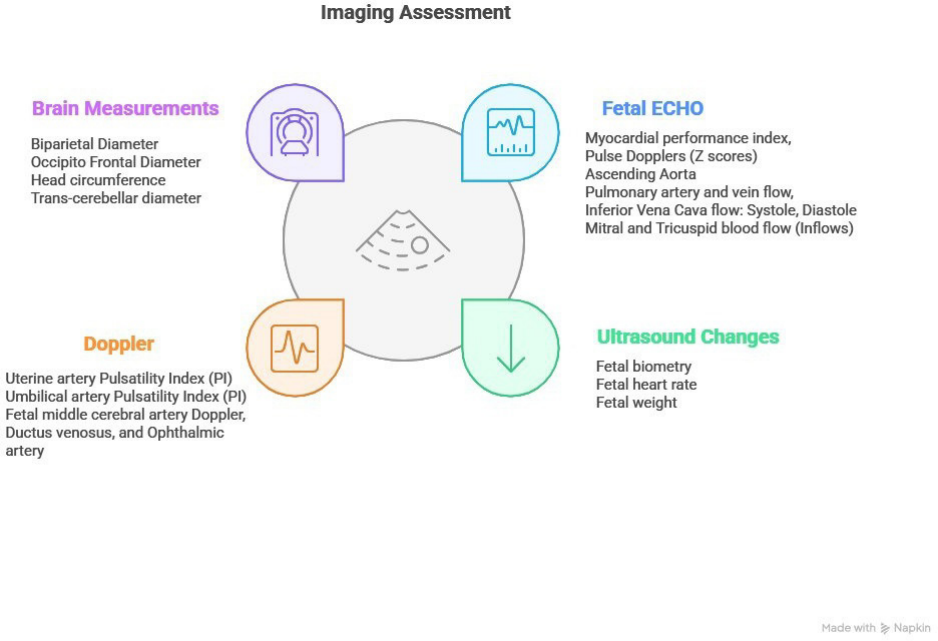
Ultrasound, Doppler and echocardiographic parameters to be assessed at scheduled antenatal visits.

To evaluate the impact of heat stress on the maternal-fetal interface, we will conduct a series of imaging studies using the Voluson Swift ultrasound system, in accordance with ISUOG guidelines. This will include dating scans, assessment of fetal viability, anomaly scans and growth evaluations scheduled at standard gestational time points (figure 2). Doppler ultrasound will assess the uterine artery, umbilical and middle cerebral artery Doppler flow using a standard protocol. Echocardiography will examine the cardiac structure and function of the fetus.

#### Environmental data collection

The Temp-U temperature and humidity logger will be worn by each participant on the upper arm inside a soft pouch supplied by the study team. They will be asked to keep the device on throughout the day for continuous recording of ambient temperature and humidity and place it beside them when they sleep. Data collection teams will visit every fortnight to download data from the Temp-U loggers. The files will be uploaded to study’s secure cloud storage, using a consistent naming format that includes the participant identification number, device type and date range. For the subcohort, Fitbit data will be retrieved every 5 days. Devices will be recharged, synchronised and returned to the participants to ensure minimal data loss. Data management system, an Airflow-based data pipeline will automatically extract cloud files, perform data cleaning and timestamp alignment and store the datasets in the Structured Query Language (SQL) Server database at the AKU Data Center. Automated alerts will be generated in case of any issue during pipeline process (eg, missing files, upload errors or transformation failures), allowing timely review and resolution. Access to the cloud folders and the central database will be restricted to authorised users. The device wear-time compliance will be monitored through spot checks and completion of device compliance forms.

#### Delivery and postnatal follow-up

A delivery notification system will be established to ensure timely recording of birth weight, with the data collection team conducting regular phone calls to pregnant women as they approach delivery. Newborn’s weight, length, mid-upper-arm circumference (MUAC) and head circumference will be recorded within 48 hours of delivery. Additionally, for the subcohort, trained clinical staff will collect placental tissue and cord blood samples within 15 min of delivery. A placental sample will be snap-frozen and stored in liquid nitrogen containers before being transported and stored at the laboratory. For future transcriptomic and epigenetic studies, serum, stool and nasopharyngeal samples from the subset of pregnant women will be collected and archived in multiple aliquots at −80°C. After delivery, the mother-infant pair will be followed for 12 months with quarterly assessments for the main cohort and monthly for the subcohort on anthropometry, and infant and young child feeding practices.

#### Anthropometric assessment

Maternal weight will be monitored throughout pregnancy and postnatally, alongside infant growth parameters following the INTERGROWTH-21st protocols.^65^ Maternal weight will be measured using a Seca 874 U electronic scale to the nearest 0.1 kg, while newborn weight will be assessed within 48 hours of birth using a Seca 354 infant weighing scale with the same precision. Recumbent length will be measured using an Infantometer, and coloured MUAC tape will be used for infants, both to the nearest 0.1 cm. Length, HC and MUAC will be recorded in cm to the last completed cm (not the nearest). All equipment will be calibrated biweekly using standardised aluminium rods for the infantometer and certified calibration weights for scale calibration. Maternal weight will be recorded twice by the same anthropometrist, while infant measurements will be recorded independently by two anthropometrists (blinded). If discrepancies exceed 0.7 cm for infants, 1.0 cm for adults (length), or 0.5 kg for children and 1 kg for women (weight), and 0.5 cm for HC, a third and final measurement will be taken and recorded independently by both anthropometrists.

### Covariates

Information will be collected on potential confounders and other variables including demographics, maternal age, socioeconomic status, parity, Body Mass Index, nutritional status, comorbidities (hypertension and diabetes), antenatal care uptake, mental wellbeing, employment history and heat exposure at each follow-up through structured questionnaires and clinical assessments. Description of the measurements of these variables is provided under the relevant subsections/headings in the data collection procedures.

### Data management and data quality

Data will be collected on a data collection application using handheld devices and transferred to the AKU server via secure internet connection. Designated personnel will periodically retrieve environmental data from wearable and installed devices, which will be returned to participants after each data transfer. All data will be archived in a secure data repository, with restricted access through AKU local area network (LAN) authentication. Ultrasound, Doppler and echocardiography scans will be centrally reviewed by a consultant sonologist and cardiologist, who will assess image quality and diagnostic accuracy. Biochemical assays will follow standardised protocols, with internal quality controls.

### Statistical analysis plan

Ambient heat exposure during pregnancy for each participant will be assessed using Mean Heat Index (HI) calculated from wearable temperature and humidity loggers using the Rothfusz regression equation, as well as Universal Thermal Climate Index (UTCI).^66^ Exposure will be examined across gestational windows relevant to fetal development for exploratory analyses. Descriptive statistics will summarise maternal characteristics, environmental exposures and birth outcomes.

For primary analyses, associations between heat exposure and adverse birth outcomes LBW and SVN will be assessed using multivariable logistic regression for binary outcomes and multivariable linear regression for continuous birth weight. Heat exposure will be modelled primarily as a continuous variable. All models will adjust for maternal age, socioeconomic status, gestational age at delivery, parity and season. Effect modification by maternal characteristics and season will be examined.

Exploratory analyses will include trimester-specific models to identify sensitive exposure windows. Sensitivity analyses excluding multiple births will assess the robustness of findings among singletons. Alternative exposure definitions will be tested for consistency, including (i) number of days with daily maximum temperature >40°C,^67^ and (ii) the 90th percentile of the temperature distribution.^68^ Exploratory analyses will evaluate additional exposure definitions to determine which metrics demonstrate the strongest associations with outcomes and could be recommended for future use or validation.

Distributed lag and time-varying exposure models will be explored to assess delayed or cumulative effects across gestation. Potential non-linear exposure–response relationships will be examined using generalised additive models (GAMs), with results reported as supplementary to the primary linear models. Given multiple exposure definitions in exploratory analyses, findings will be interpreted cautiously, focusing on effect sizes, CIs and biological plausibility rather than p values alone. For the subcohort, mediation analysis will be conducted to investigate potential biological pathways linking heat exposure to fetal growth restriction, focusing on maternal stress response, inflammation and placental dysfunction. All mediation models will adjust for key confounders. Statistical analyses will be performed using Stata version 18.

### Bias mitigation

As with any prospective cohort study, we may encounter participant attrition, which could potentially introduce selection bias. Specifically, if individuals who drop out differ systematically from those who remain (differential attrition), such as those with pre-existing health conditions, pregnancy complications or belonging to a certain socioeconomic background, it could make the remaining sample less representative of the original cohort. To minimise these biases, we will maintain frequent contact through phone calls and home visits. We will also address logistical barriers by providing free transportation to and from the study research centre and facilitating access to healthcare facilities when needed. Sensitivity analyses will help assess the impact of attrition and adjust for any potential bias. The study teams will visit at least every 5 days to check adherence, battery life and device function. To prevent loss or misuse, each sensor will be tagged with a unique identifier, and participants will receive guidance on proper use and storage.

### Ethics and dissemination

The study has received approval from the AKU Ethical Review Committee (Ref: 26249) and the National Bioethics Committee (Ref: 1065/23/1736) and is registered at ClinicalTrials.gov (Identifier: NCT01234567).

Written informed consent will be obtained from all participants, with thumbprints accepted for those unable to write. Study aims, procedures and potential risks and benefits will be explained in the local language, with opportunities for questions. Referral pathways will be established to address complications and emergencies. In case of clinical concerns (eg, high blood pressure, dehydration, pregnancy complications), participants will be referred to tertiary healthcare facilities with transportation support. Incidental findings from echocardiography or ultrasound will be communicated to the participants, and referrals will be made to specialised healthcare services.

Data confidentiality will be strictly maintained. Personal identifiers will be coded and delinked. Access will be limited to the principal investigator and project coordinator, with secure storage of both digital and printed materials. Findings will be disseminated via peer-reviewed publications, conferences and stakeholder meetings. Community engagement sessions will be conducted to share key insights with participants and local healthcare providers.
