# Supplementary material for: Biological impacts of rising temperatures on maternal, fetal and newborn health: protocol for a cohort study (BIRTH-Cohort)
Source: BMJ Open. 2026 Mar 18;16(3):e107773. doi: 10.1136/bmjopen-2025-107773 (PMC13007160; doi:10.1136/bmjopen-2025-107773)
Supplement: Supplementary file 1 [file bmjopen-2025-107773.reviewer_comments.pdf]

PEER REVIEW HISTORY

BMJ Open publishes all reviews undertaken for accepted manuscripts. Reviewers are asked to complete a checklist review form and are provided with free text boxes to elaborate on their assessment. These free text comments are reproduced below.

ARTICLE DETAILS

Title (Provisional)

Biological Impacts of Rising Temperatures on Maternal, Fetal, and Newborn Health: Protocol for a Cohort Study (BIRTH Cohort)

Authors

Das, Jai K.; Malik, Asma Abdul; Papageorghiou, Aris T; Piryani, Suneel; Ambareen, Mahnaz; zuberi, nadeem; Islam , Zaheena; Mohammed, Nuruddin; Hussain, Narjis Fatima; Samad, Zainab; Baloch, Farhala M; Qazi, Saqib; Rizvi, Arjumand; Ahmed, Imran; Iqbal, Junaid; Mohsin, Shazia Samad; Ali, Fatima; Sheikh, Lumaan; Begum, Kehkashan; Zuberi, Rabia; Cousens, Simon; Kennedy, Stephen; Bhutta, Zulfiqar Ahmed

---

VERSION 1 - REVIEW

**Reviewer**	**1**
**Name**	**Martin, Gina**
**Affiliation**	**Athabasca University Faculty of Health Disciplines, Health Disciplines**
**Date**	**15-Sep-2025**
**COI**	**None**

Thank you for the opportunity to review this study protocol. The topic is timely. I have included some recommendations for improving the clarity of the protocol.

• The introduction and theoretical linkages are clearly outlined in the protocol paper.

• The study objective could indicate the context of the study as the sample is from rural areas in Pakistan, but the objectives seem broader, so it is not clear why the sample is from rural regions. It is noted that they face extra challenges, but this isn’t addressed in the study objectives.

• The outcomes are clearly outlined, but the exposure measures are not as clearly stated in the methods. This seems to be covered in the data analysis section.

• Same comment above, but for covariates.

**Reviewer**	**2**
**Name**	**Tong, Stephen**
**Affiliation**	**Univ Melbourne, Obstetrics & Gynaecology**
**Date**	**19-Oct-2025**
**COI**	**None**

This manuscript by Das et al describes a protocol of a planned large prospective observational study to examine the potential effects of heat stress on 6000 pregnancies in Pakistan.

It is an interesting study that plans impressively deep phenotyping – from wearables monitoring heat stress and biological indices, to multiple ultrasound scans, multiple biospecimen collections and postnatal follow up.

The region that participants will be recruited from seems highly appropriate to examine the biological effects of heat - it is a hot place. Furthermore, it seems air-conditioning for most this population will be uncommon, unfortunately.

This ambitious study could yield many insights into heat stress and pregnancy. It is an exciting concept.

Major comments:

1) This study is a big exploratory analysis - ie hypothesis generating, including the analysis of the 4 primary outcomes. This may be appropriate for large cohort collections, designed to examine many things. But it means it needs to be acknowledged that findings need to be validated in future studies.

Currently, there are 4 primary outcomes. Furthermore, multiple approaches to the analysis will be done for these primary outcomes: 1) data driven analysis (which already lends itself to over-fitting and needs validation) 2) literature-based cut-offs 3) cumulative exposure metrics. Last (and see point 2 of my comments), a number of different definitions of the 'exposure' (ie heat stress) will be tested.

Hence, there isn't one definition of the exposure, there are many outcomes being studied, and there isn't one defined statistical analysis approach for the primary outcome.

Declaring this to be an exploratory study is important. With so many analyses, finding many associations with a p value <0.05 seems inevitable. Reporting them as definitive findings (especially if presented in a number of publications) risks being misleading because it could factitiously exaggerate links between heat and poor pregnancy outcomes.

If the authors want to declare findings arising from this cohort as definitive, options may be:

- Select one or two primary outcomes, show you are sufficiently powered for both, prepare an a priori statistics plan on exactly how the analysis will be done, before data unblinding. (Ie similar approach to a clinical trial). The remaining analyses are exploratory.

- Split the cohort of 6000: eg 60% as a 'discovery cohort' and 40% as a 'validation' cohort, the latter used to confirm anything found to be significant in the discovery cohort.

2) Related to my first point, a potential weakness of this study is that the definition of the exposure 'heat stress' seems not only ill defined, but the authors state it will be defined many ways.

I realise defining the exposure may be complicated. However, having different definitions of the exposure will result in an analysis that is particularly exploratory. It seems inevitable they will generate many associations with $p<0.05$, but it will be unclear which are real associations.

In general, for all analyses done of the data generated by this impressive cohort, I suggest the authors need to guard against the risk of reporting many false positives.

This could be done by diligently generating a priori statistical analysis plan for every association explored, before the statistics team are unblinded to data. And they should sensibly limit the exploratory approaches undertaken (ie be on guard against p-value 'hunting', it can inadvertently happen)

3) Can the authors justify the primary outcome of low birth weight <2500? It is an older way to define small babies that only provides very limited information because it does not take gestational age into account. It is a pragmatic clinical definition to denote a small baby for populations where gestational age is often unknown. But this study cohort will have early ultrasound determination of gestational age by the investigators.

Would the authors consider replacing this with estimated fetal weight <3rd centile (corrected for gestation) as a primary outcome instead of a secondary? This would seem a more robust indicator of placental insufficiency.

4) The fourth primary outcome seems a composite that includes the 1st and 3rd primary outcomes. Given the addition of each primary outcome weakens the significance of the other primary outcomes (ie you don't want too many primary outcomes as are then doing multiple statistical testing), it seems odd to have a primary outcome that is just a combination of other primary outcomes.

I'd consider removing this.

5) Might hypertensive disorders be added as a primary outcome (if the fourth primary outcome is taken out) given there is existing literature linking heat stress to this pregnancy complication?

6) Preterm birth <37 is appropriate, but what about preterm birth <34 weeks, even as a secondary outcome? It is a more severe preterm birth phenotype.

7) The definition of preeclampsia being hypertension and proteinuria (1+ protein) is outdated but might be appropriate in this setting. If so, I suggest the definition of proteinuria should be 2+ protein on dipstick as 1+ might risk many false positives. Ideally, the team uses hypertension and evidence of organ injury as the definition of preeclampsia. (ie ISSHP/NICE/AMJOG guidelines)

8) The authors list placental histopathology (maternal vascular malperfusion) as a secondary outcome. Do they have access to high quality placental pathology analysis to get such data on this cohort?

VERSION 1 - AUTHOR RESPONSE

Reviewer Comments

S.no.	Reviewer's Comment	Response
**Reviewer: 1**<br>Dr. Gina Martin, Athabasca University Faculty of Health Disciplines		
1.	Thank you for the opportunity to review this study protocol. The topic is timely. I have included some recommendations for improving the clarity of the protocol.<br><br>The introduction and theoretical linkages are clearly outlined in the protocol paper.	Thanks for the encouragement
2.	The study objective could indicate the context of the study as the sample is from rural areas in Pakistan, but the objectives seem broader, so it is not clear why the sample is from rural regions. It is noted that they face extra challenges, but this isn't addressed in the study objectives.	We have now added the context/ setting in all objectives<br>Line # 151-152, 154-155, 158-159 (track changes version)
3.	The outcomes are clearly outlined, but the exposure measures are not as clearly stated in the methods. This seems to be covered in the data analysis section.	We have now added the "Exposure Ascertainment" sub-heading under "Data Collection Procedures" in Methods section.<br>Line # 316-322 (track changes version)<br><br>Since the exposure (heat stress) will be calculated analytically from raw environmental measurements (daily temperature and humidity), it is described in detail in the Statistical Analysis section.
4.	Same comment above, but for covariates.	Thanks for raising this point, we have now added information on covariates and has now been described more clearly under the "covariates" subheading in Methods section.<br>Line # 408-414 (track changes version)<br><br>Details on their measurement are interspersed within the relevant subsections of the data collection

<table>
<tr><td></td><td></td><td>procedures (e.g., anthropometric assessment, delivery and postnatal follow-up, ultrasound and Doppler imaging), and removed from statistical analysis plan.</td></tr>
<tr><td colspan="3">Reviewer: 2
Dr. Stephen Tong, Univ Melbourne</td></tr>
<tr><td colspan="3">Major Comments</td></tr>
<tr><td>5.</td><td>This manuscript by Das et al describes a protocol of a planned large prospective observational study to examine the potential effects of heat stress on 6000 pregnancies in Pakistan.
It is an interesting study that plans impressively deep phenotyping – from wearables monitoring heat stress and biological indices, to multiple ultrasound scans, multiple biospecimen collections and postnatal follow up. The region that participants will be recruited from seems highly appropriate to examine the biological effects of heat - it is a hot place. Furthermore, it seems air-conditioning for most this population will be uncommon, unfortunately. This ambitious study could yield many insights into heat stress and pregnancy. It is an exciting concept.</td><td>Thank you for the detailed review and very pertinent comments</td></tr>
<tr><td>6.</td><td>This study is a big exploratory analysis - ie hypothesis generating, including the analysis of the 4 primary outcomes. This may be appropriate for large cohort collections, designed to examine many things. But it means it needs to be acknowledged that findings need to be validated in future studies.

Currently, there are 4 primary outcomes. Furthermore, multiple approaches to the analysis will be done for these primary outcomes: 1) data driven analysis (which already lends itself to over-fitting and needs validation) 2) literature-based cut-offs 3) cumulative exposure metrics. Last (and see point 2 of my comments), a number of different definitions of the ‘exposure’ (i.e., heat stress) will be tested.

Hence, there isn’t one definition of the exposure, there are many outcomes being studied, and there isn’t one defined statistical analysis approach for the primary outcome.

Declaring this to be an exploratory study is important. With so many analyses, finding many associations with a p value <0.05 seems inevitable. Reporting them as definitive findings (especially if presented in a number of publications) risks being misleading because it could factitiously exaggerate links between heat and poor pregnancy outcomes.

If the authors want to declare findings arising from this</td><td>We thank the reviewer for the insightful comments. As one of the first large-scale, prospective cohort of maternal heat exposure in Pakistan, aimed at identifying associations that have not yet been defined in this setting is inherently exploratory.

We thank the reviewer for these great points and to address the reviewer’s concerns, we have revised the manuscript as follows:

1. The study will retain two primary outcomes, low birth weight (LBW) and small vulnerable newborns (SVN), for which the sample size is powered. Other primary outcomes, preterm birth and small for gestational age (SGA) have now been moved to secondary as suggested.
Line # 202-210 and 213-219 (track changes version)

2. We have one definitive heat exposures definition and limited to the mean heat index only.
All the other alternative (post-hoc) heat stress definitions have now been explicitly labelled as exploratory.</td></tr>
</table>

	cohort as definitive, options may be:<br><br>- Select one or two primary outcomes, show you are sufficiently powered for both, prepare an a priori statistics plan on exactly how the analysis will be done, before data unblinding. (Ie similar approach to a clinical trial). The remaining analyses are exploratory.<br>- Split the cohort of 6000: eg 60% as a 'discovery cohort' and 40% as a 'validation' cohort, the latter used to confirm anything found to be significant in the discovery cohort.	Line # 426-431 (track changes version)<br><br>3. A detailed, and a priori analysis plan will be used with pre-specified analytic models.<br>Line # 423-454 (track changes version)<br><br>We hope this now suffices the concerns and
7.	Related to my first point, a potential weakness of this study is that the definition of the exposure 'heat stress' seems not only ill defined, but the authors state it will be defined many ways.<br><br>I realise defining the exposure may be complicated. However, having different definitions of the exposure will result in an analysis that is particularly exploratory. It seems inevitable they will generate many associations with $p<0.05$, but it will be unclear which are real associations.<br><br>In general, for all analyses done of the data generated by this impressive cohort, I suggest the authors need to guard against the risk of reporting many false positives.<br><br>This could be done by diligently generating a priori statistical analysis plan for every association explored, before the statistics team are unblinded to data. And they should sensibly limit the exploratory approaches undertaken (ie be on guard against p-value 'hunting', it can inadvertently happen)	In light of the reviewer comments, we now only have one exposure variable of heat as definitive<br><br>1. We will now use mean heat index as the primary exposure measures.<br>Line # 426-429 (track changes version)<br><br>2. Any alternate indices, such as total number of pregnancy days when maximum temperature exceeded 40°C, and number of days when daily max temperature exceeded the 90th percentile of the temperature distribution will be used for exploratory analyses to assess comparability with prior literature.<br>Line # 438-444 (track changes version)<br><br>3. Only the primary exposure will be used in confirmatory models for the two primary outcomes. All other analyses will be treated as exploratory. And it is important to ascertain this through different approaches as this is the overall objective of the study to assess the impact off heat and how it effects pregnancy and birth outcomes – acute or through chronic sustained effect.
8.	Can the authors justify the primary outcome of low birth weight <2500? It is an older way to define small babies that only provides very limited information because it does not take gestational age into account. It is a pragmatic clinical definition to denote a small baby for populations where gestational age is often unknown. But this study cohort will have early ultrasound determination of gestational age by the investigators.<br><br>Would the authors consider replacing this with estimated fetal weight <3rd centile (corrected for gestation) as a primary outcome instead of a secondary? This would seem a more robust indicator of	We acknowledge that estimated fetal weight adjusted for gestational age provides an accurate assessment of fetal growth restriction. However, low birth weight remains a globally recognised and policy-relevant indicator of neonatal risk, endorsed by WHO and widely used in public health, epidemiological research and climate pregnancy cohorts.<br>Given that this research intends to inform both clinical and public health interventions, LBW will remain a primary outcome along with SVN.

	placental insufficiency.	We have also explicitly included small-for-gestational-age (SGA), preterm and intrauterine growth restriction (IUGR) as secondary outcomes to complement the LBW findings.
9.	The fourth primary outcome seems a composite that includes the 1st and 3rd primary outcomes. Given the addition of each primary outcome weakens the significance of the other primary outcomes (ie you don't want too many primary outcomes as are then doing multiple statistical testing), it seems odd to have a primary outcome that is just a combination of other primary outcomes.<br><br>I'd consider removing this.	Thanks for these suggestions and we have kept LBW and small vulnerable newborns (SVN) as primary outcomes, and the sample size is powered for both.<br>The accurate first-trimester ultrasound dating will enable granular classification of SVN phenotypes, rather than using low birth weight alone, allowing much finer categorisation of neonatal vulnerability (preterm, term-SGA, preterm-SGA, etc.), which we see as a major strength of this cohort compared with most existing studies. Also, because all pregnancies are dated by first-trimester ultrasound, we can define SVN phenotypes conditional on accurate gestational age, rather than relying solely on LBW.
10.	Might hypertensive disorders be added as a primary outcome (if the fourth primary outcome is taken out) given there is existing literature linking heat stress to this pregnancy complication?	Thank you for the comment. While hypertensive disorders are an important pregnancy outcome associated with heat stress, we will not be conducting continuous blood pressure monitoring in this study. Blood pressure will be measured only at scheduled follow-up visits in the clinics (along with urine dipstick), which may limit the accuracy of exposure-outcome temporal associations. Therefore, while not a primary outcome, pre-eclampsia/ eclampsia will remain and reported as a secondary outcome in the study.
11.	Preterm birth <37 is appropriate, but what about preterm birth <34 weeks, even as a secondary outcome? It is a more severe preterm birth phenotype.	Thanks for raising this important point. We agree that distinguishing more severe forms of preterm birth provides additional insight. We will further categorise preterm birth by gestational subgroups, i.e., extreme preterm (<28 weeks), very preterm (28–31 weeks), moderate preterm (32–33 weeks), and late preterm (34–36 weeks), to allow for stratified analysis across the preterm birth spectrum.<br>Line # 213-215 (track changes version)
12.	The definition of preeclampsia being hypertension and proteinuria (1+ protein) is outdated but might be appropriate in this setting. If so, I suggest the definition of proteinuria should be 2+ protein on dipstick as 1+	Added ≥+2 to the definition of proteinuria on dipstick with reference using ISSHP guideline.<br>Line # 226 (track changes version)

	might risk many false positives. Ideally, the team uses hypertension and evidence of organ injury as the definition of preeclampsia. (ie ISSHP/NICE/AMJOG guidelines)	
13.	The authors list placental histopathology (maternal vascular malperfusion) as a secondary outcome. Do they have access to high quality placental pathology analysis to get such data on this cohort?	Yes, high-quality placental histopathology analysis will be conducted through the Department of Pathology at Aga Khan University, which has extensive expertise and established protocols for placental examination.

VERSION 2 - REVIEW

Reviewer 2

Name Tong, Stephen

Affiliation Univ Melbourne, Obstetrics & Gynaecology

Date 04-Feb-2026

COI

The authors rebuttals are thorough, clear and comprehensive. Thank you. I have no further concerns and wish the team all the best as they undertake this important study.
